# Congenital Portosystemic Shunts: Variable Clinical Presentations Requiring a Tailored Endovascular or Surgical Approach

**DOI:** 10.1097/PG9.0000000000000279

**Published:** 2023-01-12

**Authors:** Eduardo Bent Robinson, Gregory Jordan, Danielle Katz, Shikha S. Sundaram, Julia Boster, Dania Brigham, Patricia Ladd, Christine M. Chan, Rebecca L. Shay, Emily Ochmanek, Aparna Annam

**Affiliations:** From the * Department of Radiology, University of Colorado School of Medicine, Aurora, CO, USA; † Division of Pediatric Radiology, Department of Radiology, University of Colorado, Aurora, CO, USA; ‡ Division of Pediatric Gastroenterology, Hepatology and Nutrition, Digestive Health Institute, Department of Pediatrics, University of Colorado, Aurora, CO, USA; § Division of Pediatric Endocrinology, Department of Pediatrics, University of Colorado, Aurora, CO, USA; ∥ Division of Neonatology, Department of Pediatrics, University of Colorado, Aurora, CO, USA; ¶ Division of Interventional Radiology, Department of Radiology, University of New Mexico, Albuquerque, NM, USA.

**Keywords:** portosystemic shunt, hyperinsulinism, hepatic tumors, interventional radiology, endovascular closure

## Abstract

Congenital portosystemic shunts (CPSS) are rare developmental anomalies resulting in diversion of portal flow to the systemic circulation. These shunts allow intestinal blood to reach the systemic circulation directly, and if persistent or large, may lead to long-term complications. CPSS can have a variety of clinical presentations that depend on the substrate that is bypassing hepatic metabolism or the degree of hypoperfusion of the liver. Many intrahepatic shunts spontaneously close by 1 year of age, but extrahepatic and persistent intrahepatic shunts require intervention by a single session or staged closure with a multidisciplinary approach. Early detection and appropriate management are important for a good prognosis. The aim of this case series is to describe the varied clinical presentations, treatment approaches, and outcomes of 5 children with CPSS at our institution. Management of these patients should involve a multidisciplinary team, including interventional radiology, surgery, hepatology, and other medical services as the patient’s clinical presentation warrants. Regardless of clinical presentation, if a CPSS persists past 1–2 years of age, closure is recommended.

**What Is Known**

- Congenital portosystemic shunts (CPSS) are rare developmental anomalies resulting in an abnormal connection between the portal and systemic circulation.
- CPSS have a variety of presentations depending on which substrate bypasses hepatic metabolism or the degree of hepatic portal hypoperfusion.

**What Is New**

- Management of CPSS is best performed with a multidisciplinary approach that can include hepatology, surgery, interventional radiology, and other services. The approach to closure depends on shunt morphology and test occlusion pressures.
- Increased awareness of less common presentations of CPSS for clinical consideration, including imaging studies and evaluation and management options.

## INTRODUCTION

Congenital portosystemic shunts (CPSS) are rare developmental anomalies resulting in diversion of portal flow to the systemic circulation with an incidence of approximately 1:30,000 births.^1^ Embryologically, they are the result of incomplete involution of the vitelline venous system in response to the development of hepatic sinusoids, causing shunt formation.^2,3^

CPSS are typically divided into 2 categories: intrahepatic and extrahepatic. These shunts allow intestinal blood to reach the systemic circulation and, if persistent or large, may lead to long-term complications. CPSS can have a variety of clinical presentations that depend on the substrate that bypasses hepatic metabolism or the degree of hypoperfusion of the liver.^4^

There are multiple classification systems for intra and extrahepatic portosystemic shunts. Park et al^5^ have described 4 different subtypes of intrahepatic portosystemic shunts. Type I shunts have a single large vessel connecting the right portal vein to the inferior vena cava (IVC). Type II shunts are localized, peripheral shunts with one or more connections in a single hepatic segment. Type III shunts involve portal and hepatic veins connected through an aneurysm.

Type IV shunts have multiple communicating portal and hepatic veins in several segments.^5^

Morgan and Superina^6^ have developed a system to describe extrahepatic shunts, otherwise known as Abernethy Malformations, of which 2 main subtypes are described: Type I shunts (end-to-end) have a congenital absence of the portal vein resulting in complete diversion of portal blood into the IVC. Type II shunts have a hypoplastic portal vein with portal blood diverted into the IVC through a side-to-side extrahepatic communication.^6^ Neither of the classification systems can predict the clinical presentation of the shunt nor the optimal closure method.

Many intrahepatic shunts spontaneously close by 1 year of age and thus can be monitored over time if no complications arise. However, extrahepatic and persistent intrahepatic shunts require intervention by a single session or staged closure with a multidisciplinary approach, especially when associated complications are identified.^4,7^ Early detection and appropriate management are important for a good prognosis. The aim of this IRB approved case series is to describe the diverse clinical presentations, treatment approaches, and outcomes of 5 children with CPSS at our institution. The varied medical complications which can arise from CPSS, at times requiring early shunt closure, are discussed in detail below.

## CASE 1

A 34-week gestational age male infant presented with cholestasis, transient coagulopathy, hyperammonemia, hypoglycemia, respiratory insufficiency, cardiomegaly, and poor oral feeding in the newborn period. Prenatal history, prenatal imaging, and initial abdominal ultrasound without Doppler performed at another facility were unremarkable and hepatobiliary iminodiacetic acid (HIDA) scan showed delayed hepatic uptake/excretion suggestive of neonatal hepatitis.

Liver histology was consistent with canalicular and hepatocellular cholestasis with ballooning hepatocellular degeneration, with no evidence of obstructive cholangiopathy or paucity of the bile ducts.

**FIGURE 1.**
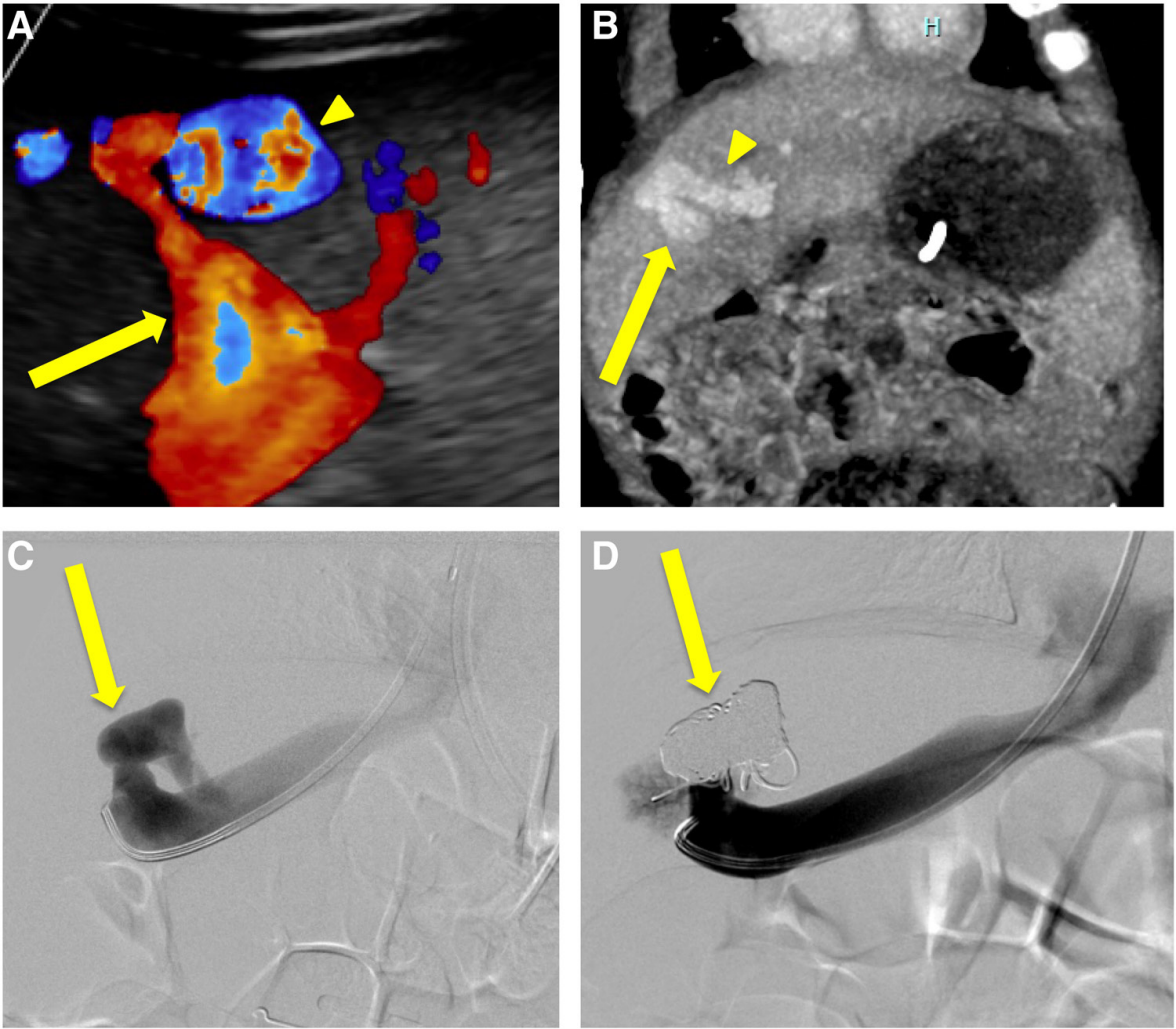
Imaging and embolization of the intrahepatic shunt. A and B) The patient’s ultrasound and CT showed an abnormal connection between the middle hepatic vein (arrow) and right portal vein (arrowhead), near the periphery of the liver. C and D) The shunt (arrow) was embolized from a transjugular approach using detachable coils. CT = computerized tomography.

The patient’s genetic and metabolic evaluations were negative, including testing for cystic fibrosis and progressive familial intrahepatic cholestasis. The infant had significant hypoglycemia, restricting the ability to consolidate feeds. Diazoxide treatment, which activates the potassium channel in the beta cell ultimately inhibiting insulin release, allowed for blood glucose stabilization following titration to a dose of 10 mg/kg/day. Repeat Doppler sonography demonstrated patent hepatic vasculature with an abnormal connection between the right portal vein and middle hepatic vein (Fig. 1A). Computerized tomography (CT) venogram confirmed an intrahepatic CPSS with a patent intrahepatic portal system and well-developed extrahepatic portal system (Fig. 1B). In the setting of persistent laboratory (Table 1) and clinical sequelae attributable to the CPSS at 43-week postmenstrual age, he was referred to Interventional Radiology for shunt closure.

**TABLE 1.** Laboratory values and liver biopsy pathology

	Preprocedure	Postprocedure
Case 1		
Ammonia (21–50 µMOL/L)	135	32
AST/ALT (13–65 U/L/12–45 U/L)	214/200	101/54
Total bilirubin (0.2–1/2 mg/dL)	5.8	1.2
Liver biopsy	Diffuse canalicular and hepatocellularcholestasis. Ballooning hepatocellular degeneration.	
Case 2		
Ammonia (21–50 µMOL/L)	114	57
AST/ALT (13–65 U/L/12–45 U/L)	629/583	196/212
Total bilirubin (0.2–1/2 mg/dL)	7.7	3.9
Case 3		
Ammonia (21–50 µMOL/L)	36	< 9
AST/ALT (13–65 U/L/12–45 U/L)	87/47	47/26
Total bilirubin (0.2–1/2 mg/dL)	0.7	0.5
Beta-hydroxybutyrate (0.02–0.27 mmol/L)	1.7	
Liver biopsy	Unremarkable liver parenchyma.	
Case 4		
Ammonia (21–50 µMOL/L)	9	9
AST/ALT (13–65 U/L/12–45 U/L)	58/30	49/27
Total bilirubin (0.2–1/2 mg/dL)	0.9	0.3
Liver biopsy	Mild sinusoidal dilation with minimal sinusoidal fibrosis. Mild microvesicularsteatosis.	
Case 5		
Ammonia (21–50 µMOL/L)	39	N/A
AST/ALT (13–65 U/L/12–45 U/L)	70/84	23/27
Total bilirubin (0.2–1/2 mg/dL)	0.5	0.3
Testosterone (prepubertal female 1–10 ng/dL)	68	7.7
T4 (thyroxine) (4.5–11.5 µg/dL)	4.6	7.7
Genetics	Negative CYP21A2 for 21-hydroxylase.	

AST/ALT = aspartate transaminase/alanine transaminase.

An endovascular, transjugular approach was used to access the CPSS via the middle hepatic vein. Given his small size and technical difficulties with available large occlusion balloons, an initial portal pressure was not obtained. Portal venograms demonstrated a robust intrahepatic portal venous system indicating he was at lower risk for acute portal hypertension. Coil embolization of the shunt was performed using multiple 4–8 mm detachable coils. Postprocedural venograms demonstrated cessation of flow through the shunt and increased filling of portal vein radicals from the right portal vein (Fig. 1C, D). Ultrasound on the following day and subsequent serial ultrasounds revealed no evidence of acute portal hypertension. The patient was able to be successfully weaned off diazoxide 1.5 months after coil embolization with long-term resolution of hypoglycemia.

## CASE 2

A 33-week small-for-gestational age female infant was delivered via cesarean section due to nonreassuring fetal heart tracing and required advanced neonatal resuscitation and intubation at birth. Her clinical presentation was notable for multifocal infantile cutaneous hemangiomas, hepatic dysfunction (elevated ammonia, aspartate transaminase/alanine transaminase (AST/ALT and bilirubin levels), and severe pulmonary hypertension requiring inhaled nitric oxide.

The presence of greater than 5 cutaneous hemangiomas prompted an abdominal ultrasound to evaluate for intrahepatic hemangiomas (Fig. 1A), which had not been seen on prenatal imaging. Ultrasound demonstrated hepatosplenomegaly and multifocal hepatic masses.

Magnetic resonance imaging (MRI) (Fig. 1B) and CT venogram confirmed multiple hepatic hemangiomas containing numerous intralesional portosystemic shunts with the largest shunt connecting the right portal vein to the right hepatic vein. She was treated with propranolol and high-dose corticosteroids to aid with involution of the hepatic hemangiomas, but her lesions did not respond to these therapies.

**FIGURE 2.**
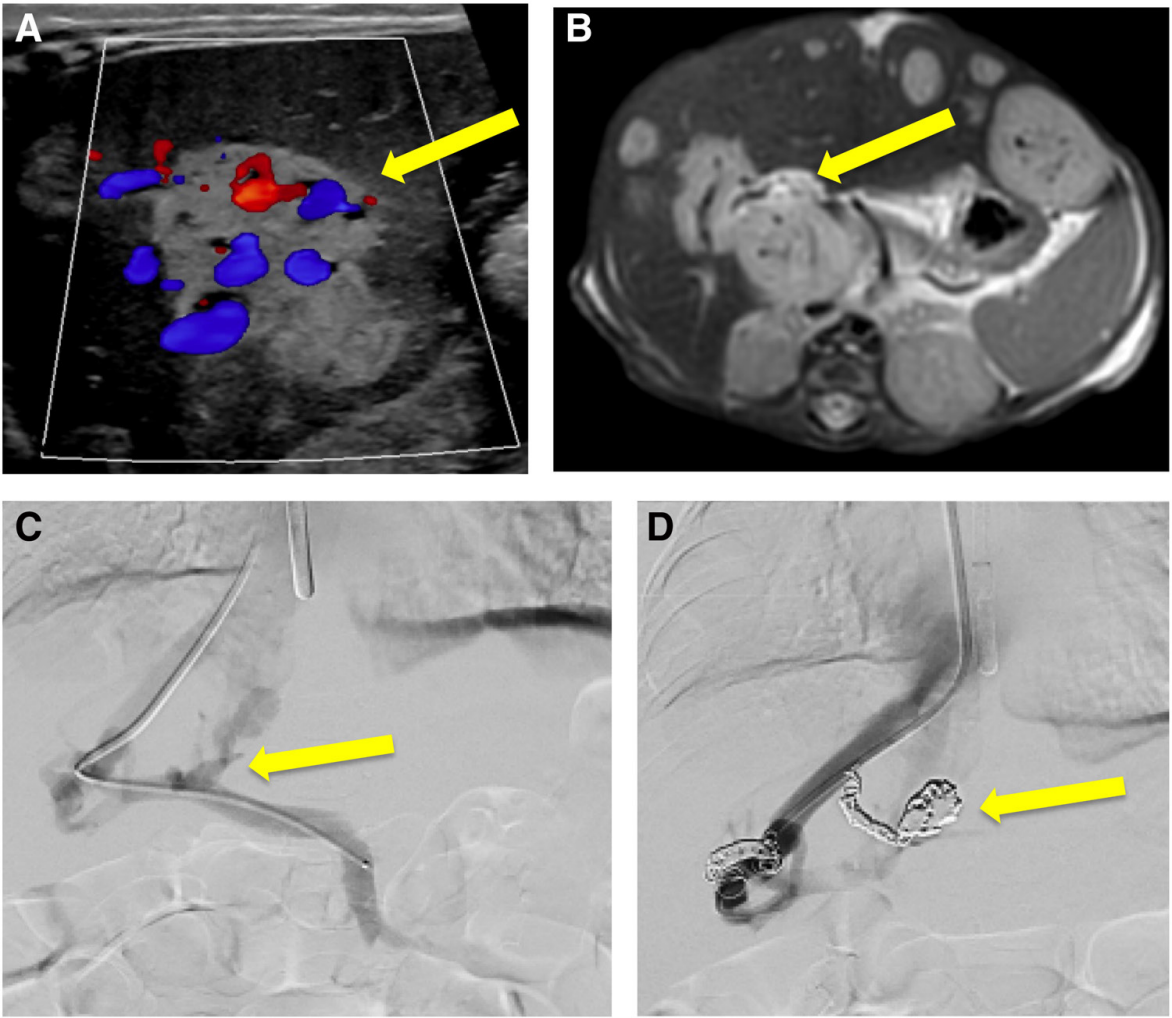
Imaging and embolization of multiple intrahepatic shunts within hepatic hemangiomas. A and B) Ultrasound with Doppler and MR imaging showed multiple hepatic hemangiomas containing portosystemic shunts (arrow). C and D) Coil embolization of four of the dominant portosystemic shunts (arrow) within hemangiomas was performed. MR = magnetic resonance.

Echocardiogram revealed a moderate patent ductus arteriosus (PDA) with right-to-left shunting. Her PDA was embolized with only a transient decrease in pulmonary hypertension. Given the concern that the portosystemic shunts were directly contributing to her pulmonary hypertension, Interventional Radiology performed portosystemic shunt mapping and embolization. Portal venography, performed at 40 weeks postmenstrual age via a transjugular approach, showed multiple central and peripheral intrahepatic shunts. Selective embolization of four of the dominant shunts was completed using multiple 3–5 mm detachable coils (Fig. 2C, D). Follow-up ultrasounds showed patent hepatic vasculature and postembolization changes within the hemangiomas but no significant decrease in the size of the hemangiomas themselves. Her postprocedural clinical course was notable for improved pulmonary hypertension by echocardiography and decreased nitric oxide requirement. Her transaminase, total bilirubin, and ammonia levels also improved (Table 1). She has had steady improvement, although not resolution, of pulmonary hypertension over the course of several months.

## CASE 3

A 2-year-old girl presented with a syncopal episode. Her previous medical history included episodes of shaking in her extremities, usually upon waking from a nap, that would improve with drinking juice. Between these episodes, she ate a regular diet and was developmentally appropriate.

**FIGURE 3.**
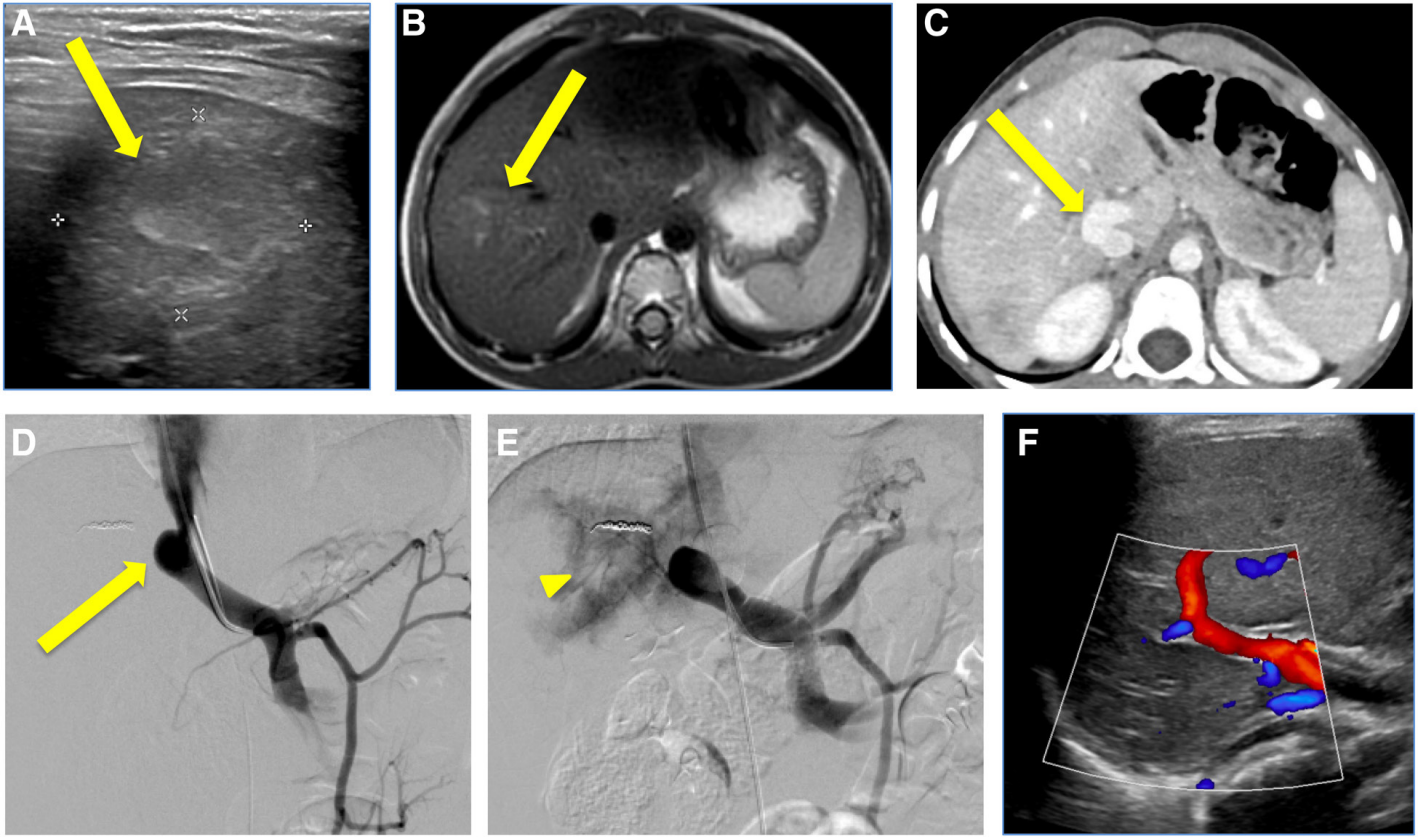
Preprocedure imaging, Interventional Radiology test occlusion venograms and post surgical follow-up imaging of the portosystemic shunt. A) Ultrasound showed an ill-defined lesion in the right lobe (arrow). B) MRI confirmed an indistinct right lobe lesion however the enhancement pattern was not specific for a benign or malignant lesion (arrow). C) CT showed an extrahepatic shunt (arrow) with a small left portal vein and a definitive right portal vein could not be detected. D, E) Venograms demonstrated a short and wide portosystemic shunt (arrow) from the diminutive left portal vein which drained directly into the inferior vena cava. Occlusion venogram showed redirected blood flow to the liver and filling of portal vein radicals (arrowhead). However the gradient was elevated and the morphology was favorable for surgical closure. F) Follow-up images show no signs of portal hypertension with good hepatopetal portal flow and resolution of the liver tumors. CT = computerized tomography; MRI = magnetic resonance imaging.

Initial laboratory evaluations revealed hypoglycemia with inappropriately high insulin levels, moderate ketosis, and mildly elevated AST/ALT levels (Table 1). An abdominal ultrasound showed an ill-defined hyperechoic right hepatic mass (Fig. 3A). A contrast-enhanced MRI confirmed a lesion in the right hepatic lobe with an indeterminate enhancement pattern, not specific for a malignant mass or a benign lesion (Fig. 3B).

Extensive testing, including endocrine and metabolic screening as well as whole exome genetic sequencing, did not identify a specific cause for the hypoglycemia. Her hypoglycemic episodes became infrequent and were managed conservatively with diet and avoidance of prolonged fasting. Surveillance imaging of the hepatic lesion was initially stable. However, after 2 years, ultrasound and MRI of the liver showed interval growth of the original lesion and development of a new enhancing lesion. At 4 years old, percutaneous biopsy of both lesions revealed normal liver parenchyma.

She presented to the emergency room one week after liver biopsy with fever, abdominal pain, and emesis. An abdominal CT angiogram showed a small pseudoaneurysm (PSA) at the biopsy site, treated with coil embolization, and a possible CPSS. Subsequent CT venogram demonstrated a large shunt between the infrahepatic IVC and the main portal vein, which had not been apparent on prior imaging due to location of the shunt and contrast timing (Fig. 3C).

Interventional Radiology evaluated the shunt for optimal closure. The IVC was catheterized with an occlusion balloon by a transfemoral approach, while the CPSS was accessed by a transjugular approach. Venograms demonstrated a large, wide neck portocaval shunt (Fig. 3D). Venograms performed with balloon occlusion showed few small intrahepatic portal vein radicals and the portosystemic shunt pressure gradient increased from 8 to 22 mm Hg (Fig. 3E). Given the short and wide morphology of the shunt and large change in pressure gradient with test occlusion, this CPSS was deemed suboptimal for endovascular closure. The patient was referred to pediatric surgery for a staged portosystemic shunt ligation (Table 2).

**Table 2.** Shunt evaluation and balloon test occlusion results

	Case 3	Case 4
Nonoccluded (mm Hg)	7–8	6
Occluded (mm Hg)	19–22	11–14
Gradient (mm Hg)	11–15	5–8
Location	Extrahepatic	Intrahepatic
Morphology	Short wide	Long narrow
Method of closure	Staged surgical ligation	Endovascular

During surgery, a trial of complete portosystemic shunt occlusion resulted in intraprocedural elevated portal pressures up to 30 mm Hg with associated bowel discoloration and congestion consistent with acute portal hypertension. Therefore, a partial shunt ligation was performed, and a catheter was left in a branch of the superior mesenteric vein for ongoing portal venous pressure monitoring. After 5 days, the portal venous pressure dropped to 14 mm Hg and a complete surgical ligation was performed without ongoing concerns of bowel congestion. She was discharged home in stable condition. Hypoglycemia has since resolved, and the hepatic lesions are no longer seen on imaging (Fig. 3F).

## CASE 4

A 5-year-old boy presented with mild developmental delay and long-standing malnutrition with a body mass index (BMI) z-score of −3.82. Birth history was significant for omphalitis, a known precipitating factor for portal vein thrombosis with cavernous transformation.^8^ An extensive gastroenterology evaluation demonstrated slightly elevated liver transaminase levels and mild hyperammonemia, and a liver biopsy showed minimal sinusoidal fibrosis and steatosis (Table 1).

**FIGURE 4.**
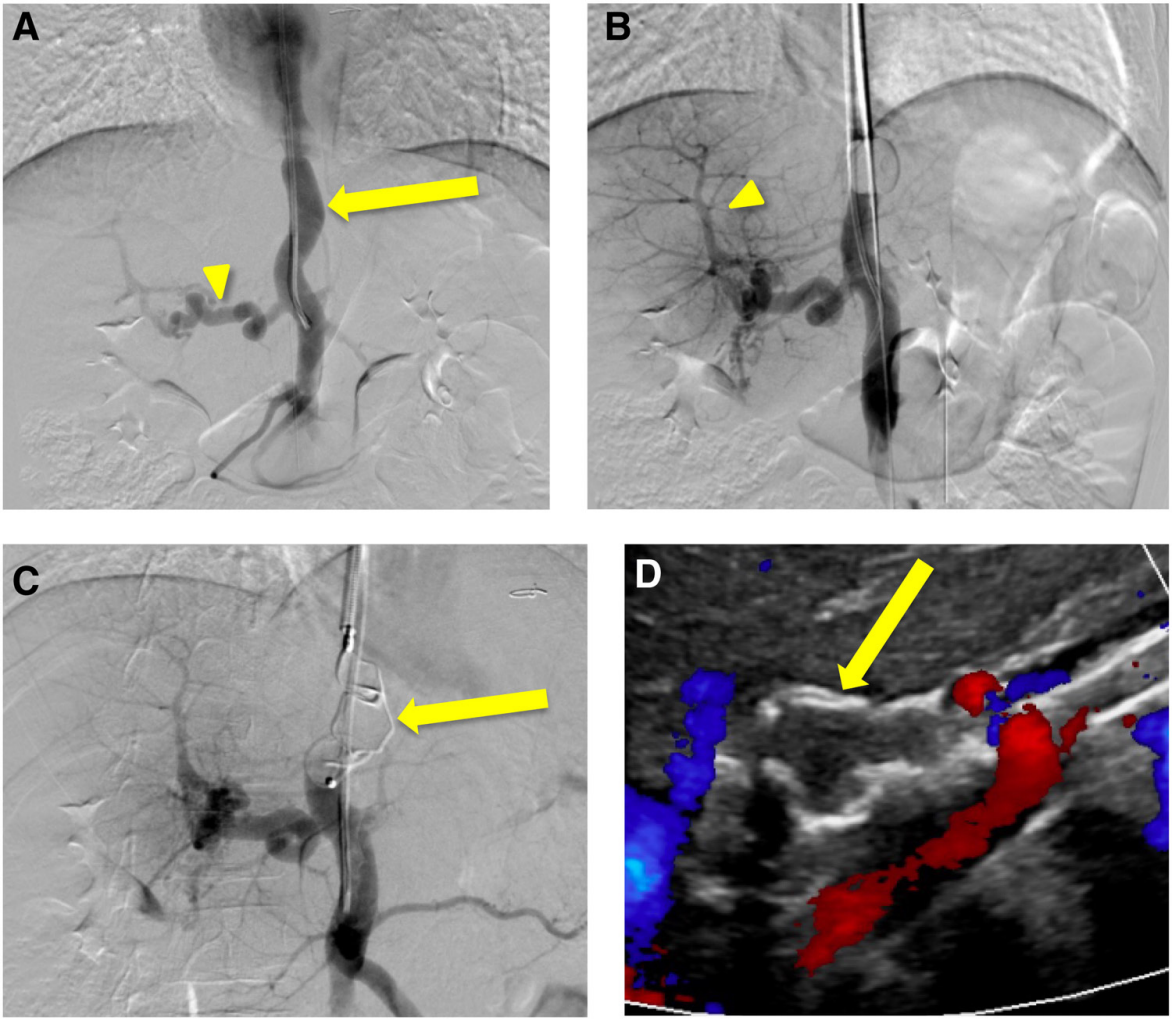
Interventional Radiology test occlusion venograms, embolization and follow-up imaging of the portosystemic shunt. A) Venograms demonstrated cavernous transformation of the main portal vein (arrowhead) and a long and narrow intrahepatic portosystemic shunt (arrow) connecting the superior mesenteric vein/splenic vein confluence with the left hepatic vein. B) Occlusion venogram showed multiple intrahepatic portal vein radicals (arrowhead). C) A 16-mm Amplatzer 2 plug (arrow) was deployed at the proximal aspect of the shunt. D) Follow-up ultrasound imaging shows complete occlusion of the shunt with the Amplatzer 2 plug (arrow) and preservation of flow within the left hepatic vein and left portal vein.

Further diagnostic testing was performed with a CT abdomen/pelvis and ultrasound with Doppler that showed cavernous transformation of the main portal vein and an intrahepatic CPSS with a prominent vessel connecting the superior mesenteric/splenic vein confluence with the left hepatic vein. Transjugular portal venography with test occlusion confirmed a long and narrow shunt that drained into the left hepatic vein and a dominant tortuous vessel representing the main portal vein (Fig. 4A). Test balloon occlusion showed multiple intrahepatic portal vein radicals and revealed a pressure gradient of 5–8 mm Hg with an absolute occlusion pressure of 11–14 mm Hg (Fig. 4B). The morphology, portosystemic shunt gradient and absolute occluded portal venous pressure were optimal for endovascular closure (Table 2).

Interventional radiology successfully deployed a 16-mm Amplatzer 2 plug into the shunt (Fig. 4C). Post procedure venogram showed increased flow to the liver and subsequent ultrasounds with Doppler showed no evidence of residual flow around the occlusion device or signs of portal hypertension (Fig. 4D). Clinically, the patient has been gaining weight with normalization of his liver function tests.

## CASE 5

A 6-year-old girl presented with precocious puberty, including acne vulgaris, axillary/pubic hair, and tall stature (99th percentile for height). Initial evaluation with endocrinology was suspicious for gonadotropin-independent precocious puberty. She had an advanced bone age of approximately 8–10 years of age. Laboratory evaluation revealed an elevated testosterone level of 46.7 ng/dL (normal for Tanner Stage II 7-28) with normal levels of androstenedione, 17-OH progesterone, and dehydroepiandrosterone sulfate (DHEAS). Her thyroxine level was borderline low (Table 1).

**FIGURE 5.**
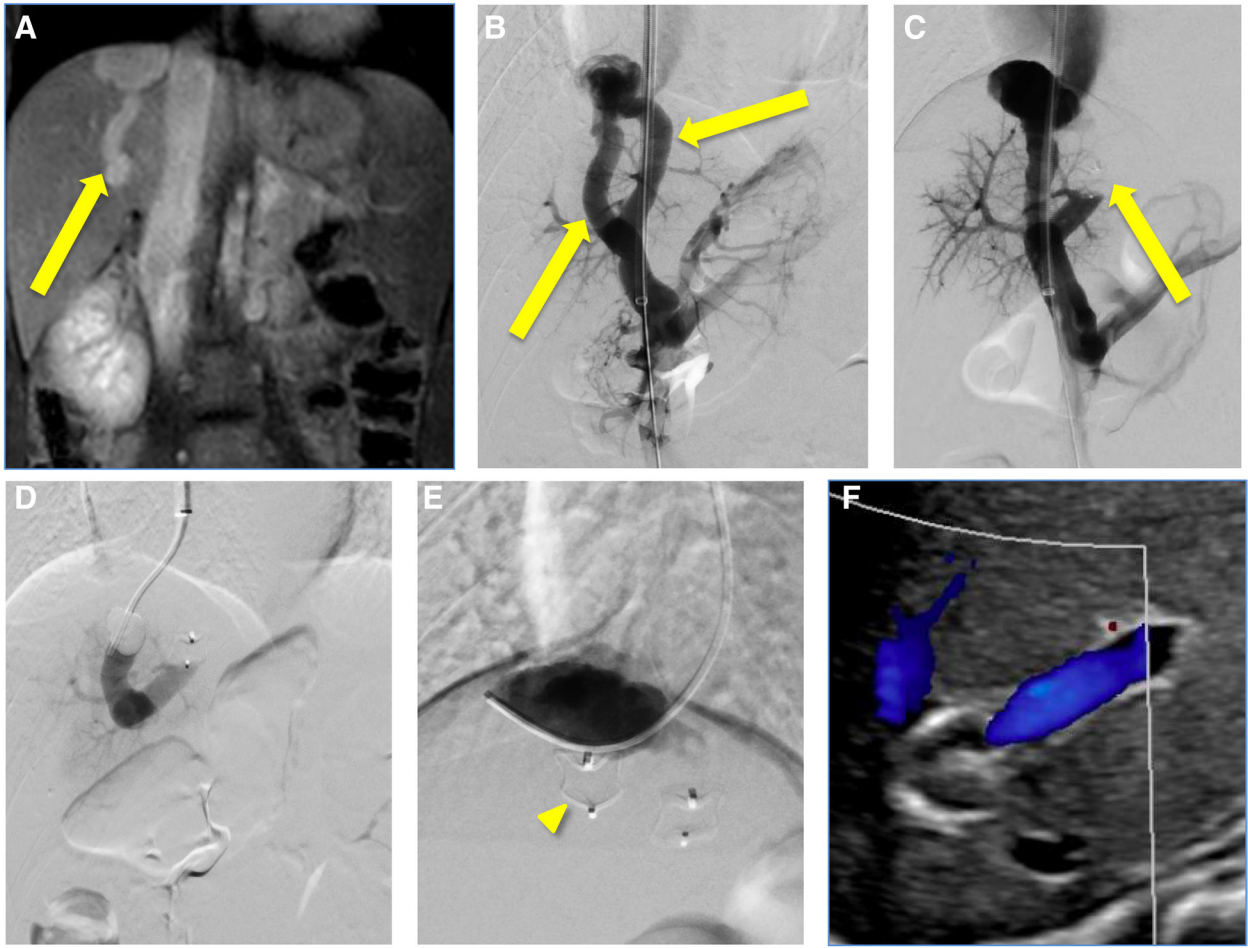
Preprocedure imaging, Interventional Radiology test occlusion venograms, embolization and follow-up imaging of the portosystemic shunts. A and B) Coronal MR image and initial venogram demonstrated two intrahepatic shunts (arrows) connecting the main portal vein to the IVC with aneurysmal drainage at the hepatic dome. C) The first shunt was occluded using a 12-mm Amplatzer 1 plug (arrow) deployed from a transjugular approach. D) Test occlusion of the second shunt performed with a Fogarty balloon did not show a significant increase in portal pressure. E) The second shunt was then occluded using a 14-mm Amplatzer 1 plug (arrowhead). F) Follow-up imaging showed occlusion of both shunt. IVC = inferior vena cava; MR = magnetic resonance.

She was evaluated for malignant abdominal/pelvic processes and congenital adrenal hyperplasia (CAH), including a normal ACTH stimulation test and negative genetic testing for CYP21A2 gene mutations. CYP21 A2 encodes 21-hydroxlyase enzyme and 21-hydroxylase deficiency is the most common cause of CAH. Pelvic ultrasound was negative for ovarian pathology. An abdominal MRI confirmed no adrenal masses but showed 2 intrahepatic CPSS connecting the main portal vein to the IVC via an enlarged hepatic vein confluence at the hepatic dome (Fig. 5A).

Hepatic transaminase levels (AST/ALT) were mildly elevated; the ammonia level was normal. Both intrahepatic shunts were occluded via a transjugular approach using 12- and 14-mm Amplatzer 1 plugs. Postocclusion portal venography showed an increased prominence of portal radicals including a large radicle, likely representing the right portal vein, and no visualization of a left portal vein (Fig. 5B–E). Postprocedure ultrasound demonstrated complete occlusion of the shunts with patent hepatic vasculature (Fig. 5F). No evidence of portal hypertension was seen on subsequent ultrasound evaluations. The testosterone level was normalized, and pubertal development and growth velocity normalized for age.

## DISCUSSION

CPSS can have a variety of clinical presentations. Some patients experience hypoglycemia due to a large quantity of insulin delivery bypassing hepatic first pass metabolism and shunting into systemic circulation, resulting in hyperinsulinism.^4,9^ Altered fetal vascular hemodynamics have been found to cause hepatic hypoperfusion, cholestasis, and fetal intrauterine growth restrictions in the absence of other biochemical, genetic, or infectious processes.^10^

Intrahepatic vascular malformations can be complex and may even obscure the presence of CPSS. The differential for macrovascular shunts includes infradiaphragmatic total anomalous pulmonary venous return, arteriovenous malformations, infantile hepatic hemangiomas, arterioportal fistula, and portosystemic shunts.^11^ Large or multiple hepatic hemangiomas can have an increased risk of high vascular flow resulting in hemodynamic instability and heart failure.^12^ Pulmonary hypertension in neonates can be associated with congenital portosystemic shunts. The pathophysiology is incompletely understood, but it is thought to be secondary to fluid overload as well as a lack of hepatic clearance of vasoactive substances.^12^ In some patients with CPSS, shunt closure or liver transplantation may prevent the progression of pulmonary hypertension.^13^

Hepatic tumors can develop from altered hemodynamics secondary to insufficient portal vein perfusion and compensatory increased hepatic arterial flow. This can lead to elevated levels of hepatic growth factors (insulin, glucagon, hepatocyte growth factor).^4,14^ A wide range of tumors can develop ranging from benign to malignant^15^ that are more commonly seen in patients with extrahepatic CPSS. Once normal liver perfusion is established, these tumors often resolve.^4^

Hyperammonemia, as well increased levels of other neurotoxins that are not able to be measured, can cause developmental delay and growth failure. CPSS can result in shunting of portal blood away from the liver causing nitrogen-containing compounds, such as ammonia, to enter the systemic circulation.^16,17^ This can manifest as subclinical encephalopathy.

Chronic liver disease from hepatic hypoperfusion may also result in long-term cognitive effects.^4,18^ While our fourth patient in this series was known to have parents with short stature, additional factors may have contributed to his poor growth. It is hypothesized that decreased portal flow to the liver can result in diminished insulin metabolism by the liver and subsequently decreased levels of insulin-like growth factor binding protein (IGFBP-1) and decreased activity of insulin growth factor (IGF-1). This ultimately is thought to result in a state of growth hormone (GH) resistance and reduced muscle mass.^19^

An extremely rare presentation of CPSS is precocious puberty.^20,21^ The shunt itself is easily identifiable with cross-sectional imaging during evaluation for adrenal tumors.

Hyperandrogenemia in patients with CPSS occurs secondary to impaired hepatic sulfation of DHEA to its less active form DHEAS because of the bypass of DHEA into systemic circulation.^21,22^ The production of more potent downstream androgens is upregulated by increased levels of DHEA in the setting of normal or low DHEAS, which results in premature adrenarche.^22^

Characterization of the CPSS morphology and flow dynamics during test balloon occlusion aid in determining the closure approach. Test occlusion of the CPSS evaluates for changes in portal pressure measurements to determine feasibility of single session closure, either surgical or endovascular, versus a need for staged surgical approach. Single session closure is pursued if test occlusion results in a portal pressure gradient <10 mm Hg and the absolute occluded portal pressure remains <25 mm Hg. If these parameters are not met, a staged surgical occlusion may be preferred. The morphology of a short wide neck shunt lacks an optimal landing zone for endovascular closure devices and may be more amenable to surgery (Table 2).^23^

This case series helps to highlight the various potential presentations, evaluation, and approaches to shunt closure. Early shunt closure should be considered when significant complications arise from CPSS, including hypoglycemia, hyperammonemia, pulmonary hypertension and precocious puberty, among other clinical manifestations. None of the patients in our series experienced complications attributable to shunt closure, such as portal hypertension, and endovascular balloon occlusion testing of the shunt can be helpful in predicting risk of portal hypertension with CPSS closure. Management of these patients should involve a multidisciplinary team, including interventional radiology, Surgery, Hepatology and other medical services as the patient’s clinical presentation warrants. Regardless of clinical presentation, if a CPSS persists past 1–2 years of age, closure should be considered to prevent long-term sequelae.
